# Failure to detect the 22q11.2 duplication syndrome rearrangement among patients with schizophrenia

**DOI:** 10.1186/1744-9081-4-10

**Published:** 2008-02-19

**Authors:** Anna Brunet, Lluís Armengol, Trini Pelaez, Roser Guillamat, Vicenç Vallès, Elisabeth Gabau, Xavier Estivill, Miriam Guitart

**Affiliations:** 1 Genes and Disease Program, Barcelona Genotyping Node, CeGen-CRG, CIBER en Epidemiología y Salud Pública (CIBERESP), Center for Genomic Regulation (CRG-UPF), Barcelona, Catalonia, Spain; 2 Genetic laboratory UDIAT-Centre Diagnòstic, Department of Mental Health Department of Paediatrics, Fundació Parc Taulí – Institut Universitari UAB, Corporació Sanitària Parc Taulí, Sabadell, Catalonia, Spain; 3 Unitat d'Antropologia Biològica, Departament de Biologia Animal, Biologia Vegetal i Ecologia, Universitat Autònoma de Barcelona, Cerdanyola del Vallès, Catalonia, Spain; 4 Department of Mental Health, Consorci Sanitari de Terrassa, Terrassa, Catalonia, Spain; 5 Department of Health and Experimental Life Sciences, Pompeu Fabra University (UPF), Barcelona, Catalonia, Spain

## Abstract

Chromosome aberrations have long been studied in an effort to identify susceptibility genes for schizophrenia. Chromosome 22q11.2 microdeletion is associated with DiGeorge and Velocardiofacial syndromes (DG/VCF) and provides the most convincing evidence of an association between molecular cytogenetic abnormality and schizophrenia. In addition, this region is one of the best replicated linkage findings for schizophrenia. Recently, the reciprocal microduplication on 22q11.2 has been reported as a new syndrome. Preliminary data indicates that individuals with these duplications also suffer from neuropsychiatric disorders. In this study we have investigated the appropriateness of testing schizophrenia patients for the 22q11.2 microduplication. We used multiplex ligation-dependent probe amplification (MLPA) to measure copy number changes on the 22q11.2 region in a sample of 190 patients with schizophrenia. Our results corroborate the prevalence of the 22q11.2 microdeletion in patients with schizophrenia and clinical features of DG/VCFS and do not suggest an association between 22q11.2 microduplication and schizophrenia.

## Findings

Low copy repeats (LCRs) on chromosome 22q11.2 mediate chromosomal rearrangements leading to recurrent deletions, duplications and translocations [1]. The 22q11.2 microdeletion is responsible for the DiGeorge and Velocardiofacial Syndromes (DG/VCFS) (OMIM #188400 and #192430). These syndromes are associated with variable phenotypic features that includes cardiac defects, palate anomalies, characteristic physiognomy, learning difficulties and relatively high frequency of severe mental illness like schizophrenia. The majority of human chromosome 22q11.2 microdeletions (87%) are ~3 Mb in size, whereas a small proportion (8%) involve smaller nested 1.5 Mb microdeletion [2]. The reciprocal microduplication has been reported in several individuals [3]. This new chromosomal syndrome was identified in patients ascertained on the basis of to DG/VCFS-like features and up to 48 cases of the 22q11.2 microduplication have been reported [4]. The clinical presentation of 22q11.2 microduplication cases is highly variable, ranging from severe congenital malformations that lead to early death, to isolated mild learning disabilities. Most of the reported individuals are infants or children at present, in who the existence of neuropsychiatry disorders has not yet been fully assessed. However, preliminary data show that individuals with duplications also suffer from neuropsychiatric disorders. Furthermore, characteristic traits of impulsivity, aggression, oppositional defiant disorder, social immaturity, short attention spans, attention deficit disorder, and cognitive deficits have been reported in cases of 22q11.2 microduplication [3,5-8].

Chromosome 22q11.2 region provides the most convincing evidence of an association between molecular cytogenetic abnormality and schizophrenia.

Although 22q11.2 microdeletion occurs in the population at a rate of 0.016%, it has been found in up to 2% of adult schizophrenic patients and in up to 6% of cases of early onset schizophrenia [9,10]. Several reports suggest that dosage changes in 22q11.2 genes could disrupt processes required for proper brain development and/or function, and contribute to increase schizophrenia susceptibility [11,12]. Several studies have suggested linkage between 22q11 and schizophrenia [13-15] and it is one of the best replicated linkage findings among schizophrenia patients. Meta-analysis of genome scans for bipolar disorder and schizophrenia have also identified chromosome 22q11-13 as one of the strongest linkage regions for both syndromes [16].

These linkage findings indicate that mutations of genes on 22q11 are likely to contribute to susceptibility to schizophrenia. In the same direction association studies have reported several genes of this region associated with risk to develop schizophrenia and bipolar disorder, including *PRODH*, *COMT*, *ZNF74*, *PCQAP*, *UFD1L*, *ZDHHC8*, *DGCR2 *and *SNAP29 *[17,18]. In addition, neuroimaging studies revealed that 22q11.2 deletion patients exhibit a pattern of cortical gray matter reduction similar to schizophrenic subjects [19]. Finally, mouse models provide evidences that this region is associated with schizophrenia. A murine model overexpressing the mouse orthologs of several genes in this region (*CDCrel*, *GP1Bβ*, *TBX1 *and *WDR14*) exhibits behavioral abnormalities consistent with schizophrenia traits [20].

It seems extremely likely that the 22q11.2 region harbours genes that alone, or in combination, could be causally implicated in schizophrenia. Nowadays the phenotype of individuals with 22q11.2 microduplication shows a wide range of severity but some consistent findings have been dysmorphic features, cognitive deficits and psychiatric disorders reported in 33% of cases [6]. In this study we decided to investigate the appropriateness of testing schizophrenia patients for 22q11.2 microduplication. We used multiplex ligation-dependent probe amplification (MLPA) to measure copy number changes on 22q11.2 region in a set of patients with schizophrenia.

A total of 190 Caucasian, unrelated patients with schizophrenia (153 men and 40 women; age 33,3 ± 8,6 years) were included in the study. Educational levels were divided into the following categories: 54% of patients have primary school (1–11 years), 26% secondary school (12–16 years), 7% high school (17–18 years), 7% university level and finally 6% can only read and write. Patients were diagnosed according to DSM-IV criteria as follow: 126 paranoid, 14 disorganized, 13 undifferentiated, 9 residual, 13 schizoaffective disorder, 13 schizophreni form disorder and 2 cases were not specificated. All cases have been recruited at the department of mental health of two hospitals, from Sabadell and Terrassa, after written informed consent approved by the ethics board of the Fundació Parc Taulí Health Corporation.

A set of MLPA probes to screen copy number changes in the 22q11.2 region was developed according to the procedures described elsewhere [21]. The set consists of 30 different oligonucleotides that correspond to 15 different probes, 4 of which interrogate the 3 Mb region that is typically deleted in cases of DG/VCFS and the remaining 11 probes match to other regions of chromosome 22 and to other autosomal region on chromosome 10 (Table 1).

**Table 1:** Probes, genes and MLPA outcome in the analysis of the 22q11.2 duplication syndrome rearrangement among patients with schizophrenia

Probe	Location	Gene	Chrom	Start	End	Length (nt)	MLPA result
HIRA	22q11.21	HIRA	22	17698971	17699021	93	*Del (EZ-117 and EZ-238)*
TBX-1	22q11.21	TBX1	22	18150824	18150883	104	*Del (EZ-117 and EZ-238)*
COMT	22q11.21	COMT	22	18335516	18335575	110	*Del (EZ-117 and EZ-238)*
ZDHHC8	22q11.21	ZDHHC8	22	18513602	18513661	113	*Del (EZ-117 and EZ-238)*
RP11-307O16	22q11.22		22	20838313	20838371	101	Dup (EZ-18)
RP11-722K16	22q11.22	IGLC1	22	20999611	20999670	132	Dup (EZ-149)
RP11-757F24	22q11.22	IGLC1	22	21157367	21157421	97	
RP11-281O23	22q11.22	IGLC1	22	21292358	21292410	95	
RP11-50L23	22q11.22	IGLC1	22	21439794	21439859	124	Dup (EZ-6)
RP11-264C20	22q11.22	IGLC1	22	21526749	21526796	90	Dup (EZ-6), Del (EZ-105)
RP11-165G05	22q11.22		22	21655494	21655558	107	Dup (EZ-6)
CARD10	22q13.1	CARD10	10	36236312	36236371	128	
CXCL12	10q11.23	CXCL12	10	44192388	44192461	116	Del (EZ-96 *and *EZ-125)
C10orf10	10q11.23	RASSF4 C10orf10	10	44792765	44792844	122	
ZWINT	10q21	ZWINT	10	57789521	57789580	120	

Del, deletion; Dup, duplication; EZ, schizophrenia sample.

**Figure 1:**
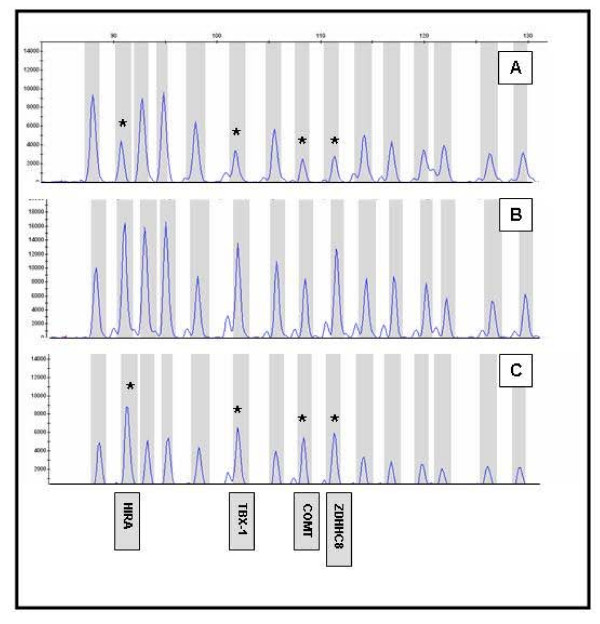
**MLPA electropherograms showing the peaks from the probes set used in the analysis of the chromosome 22q11.2 duplication syndrome.** Specific probes for the chromosome 22q11.2 region (*HIRA*, *TBX-1*, *COMT *and *ZDHHC8*) are indicated with arrows. **A**: A 22q11.2 microdeletion trace. **B**: A normal sample trace. **C**: A 22q11.2 microduplication trace.

One hundred and sixty patients were analyzed with this mix of probes. Another group of 30 individuals were also analyzed using the commercial SALSA P023 MLPA-DiGeorge syndrome test kit (MRC-Holland, Netherlands). This commercial kit consists of 39 cloned probes that interrogate different chromosomal regions associated to DG/VCFS and Cat Eye Syndrome. In this case, the microdeletion region is covered by seven specific probes and a subtelomeric 22q13 control probe. All our MLPA experiments contained the corresponding positive controls (DNA from individuals previously diagnosed as carriers of 22q11.2 duplications or deletions) to ensure the reliable detection of copy number gains and loses (Figure 1).

The MLPA reactions were performed essentially as described by Schouten et al. [22]. For the data analysis we calculated the relative probe signals using the peak heights of PCR products. Briefly, the tracing data was normalized by dividing each probe's peak height by the average height of all peaks of the sample and then dividing this value by the average normalized peak's height of the corresponding locus of all the samples. The product of this calculation is termed dosage quotient (DQ). A calculated DQ value below 0.65 was considered as indicative of a deletion, and values above 1.3 are indicative of duplications.

Among the 190 schizophrenic patients analyzed, we identify two cases (EZ-117 and EZ-238) with the 22q11.2 microdeletion. This incidence of about 1% is in agreement with previous estimates in the literature [23,24]. We did not detect the reciprocal 22q11.2 microduplication. The deleted samples were analyzed with both the in-house and commercial MLPA assays, with similar DQ values below the 0.65 threshold. A confirmatory FISH analysis was also performed (data not shown). Both patients showed common manifestations associated with DG/VCFS, including malformations of the cardiovascular system (aberrant origin of subclavian artery/pulmonary stenosis), facial dysmorphic features (long face, small ears, narrow palpebral fissures, prominent tubular nose), velopharingeal insufficiency with severe hipernasality, motor delay, cognitive deficit and mild mental retardation. These two patients with the 22q11.2 microdeletion were girls that were noted to have history of learning and behavioural problems in the school. Both cases are diagnosed as hebephrenic schizophrenia with early age of onset (12–13 years).

Subsequent family analyses in the parents showed the 22q11.2 microdeletion in the mother of one patient. The mother with same facial appearance than her daughter showed paranoid schizophrenia and mild mental retardation. This family has another offspring, a boy who was diagnosed with attention-deficit hyperactivity disorder (ADHD) during childhood. He proved to be normal by FISH analyses (not shown).

Our study indicates that MLPA is a suitable, easy, rapid and cost-effective method to seek for copy number variations (microdeletions and microduplications) in patients suffering from psychiatric disorders. Furthermore, our results confirm the prevalence of the 22q11.2 microdeletion in patients with schizophrenia and other clinical features of DG/VCFS, but do not indicate any association with the 22q11.2 microduplication. We conclude that screening for 22q11 microduplication in patients with schizophrenia is not indicated.

## Abbreviations

LCR**s**: Low copy repeats, DG/VCFS: DiGeorge and Velocardiofacial Syndromes, MLPA: Multiplex ligation-dependent probe amplification, FISH: Flourescence in situ hybridization, ADHD: Attention-deficit hyperactivity disorder

## Competing interests

The author(s) declare that they have no competing interests.

## Authors' contributions

ABparticipates in the design of the study, carried out the molecular genetic studies and drafted the manuscript. LLAparticipate in the design of the study and helped to draft the manuscript. TPcontributed with samples collection of patients and clinical characterization. RGand VVmembers of the Psychiatric Genetics Network Group participated in the conception, design of the study; data analysis and drafting of the manuscript. EGcontributed with clinical characterization of patients with 22q11.2 microdeletion. XEand MGparticipated in the conception, design and coordination of the study; data analysis and drafting of the manuscript.

All the authors read and approved the final manuscript.
